# Surgical Operations and the Workmen's Compensation Act

**Published:** 1913-03-29

**Authors:** 


### LEGAL NOTES FOR INSTITUTIONAL WORKERS.

(The Editor will be pleased to answer Legal queries in this column.)

### Surgical Operations and the Workmen's Compensation Act.

Many medico-legal questions have had their origin in the Workmen's Compensation Act, and none of them is so perplexing as that which we propose to notice in this article—the effect of a workman's willingness, or refusal, to undergo an operation on his right to compensation. In practice the question arises thus: The workman who receives injury by accident arising out of, and in the course of, his employment is awarded compensation and continues to receive half his wages so long as his incapacity continues. But it is open to the employer at any time to apply to have the compensation ended, and if he prove that the workman's incapacity has ceased, the application will be granted. The employer may also have cause to believe that, though the incapacity has not ceased, yet it would cease if not immediately, yet very soon, if the workman underwent an operation. The workman may say to such a suggestion: "I do not care to run the risk of an operation; I am in bad health, and do not wish to face it;" or "I am not bound to undergo an operation for the convenience of my employer; I prefer to draw my half wages and remain incapacitated." To such an attitude the Courts have laid down that if the workman's refusal to undergo an operation is unreasonable, his incapacity will no longer be attributed to the original accident in respect of which compensation was awarded, but to his own unreasonableness, and the payment of the compensation money will accordingly be ended.

### Is the Refusal Reasonable?

It is just here, however, that the difficulty comes in—how to determine when the workman's refusal is reasonable or unreasonable; and we shall briefly mention a recent decision of the First Division of the Court of Session (*O'Neill* v. *John Brown & Co.*, 1913, 1 S.L.T. 211), both in order to bring out the point decided and also because in their opinions the judges reviewed the previous decisions on the matter.

All the cases fall to be determined, each upon its own individual circumstances, and, indeed, the introduction into the principle above mentioned of the word "reasonable" makes that clear, because a refusal may be perfectly reasonable in one set of circumstances and equally unreasonable in another. If the operation involves a grave amount of risk to the patient the Court will not consider the workman as bound to undergo it. The case of *Tutton*, 1909, 2 K.B. 54, is an instance of that principle. There, according to the evidence, which the Court believed, of the workman's own doctor, he had either to face a very serious operation without an anæsthetic, or a very grave risk of succumbing under an anæsthetic. He was suffering from Bright's Disease. In these circumstances the Court held that the workman justifiably refused to undergo an operation.

Another principle deducible from the decisions is that if an operation is not necessary for the restoration of capacity it will not be forced on the workman. Thus in *Sweeney*, 5 F. 72, the Court, on the opinion of an eminent professor that the man was mending and might recover without any operation at all, found that he was reasonable in refusing to be operated upon for the purpose merely of accelerating the date at which the compensation would terminate.

### Eccentric Medical Opinions.

Now in the case of *O'Neill*, already referred to, the facts were that the operation was admittedly simple and unattended with risk, and the workman was well and strong. He averred that he personally was willing to undergo the operation, but two qualified medical men advised him not to undergo it as it would not lessen his incapacity in any way, and he therefore refused to submit to it. Was such refusal reasonable? Counsel for the workman strove to establish the proposition that if a man *in bona-fide* refuses to undergo an operation upon the advice of a qualified medical practitioner that must be taken as a complete answer to any suggestion that he is unreasonable in refusing to undergo it. The Court declined to countenance such a doctrine, for "if such were laid down this part of the Act would really become a dead-letter, because in the wide world of medicine it would almost always be possible to obtain a perfectly genuine, though eccentric, opinion from some qualified medical man to any effect that might be desired, within limits."

### The Test of Public Opinion.

Proceeding to examine the facts of the case before them, the Court found that all the medical men concerned, those advising the workman (two in number) as well as those advising the employer (three in number), were agreed that the operation would not be attended by any risk or danger, and at the worst no material harm could come of it, though the workman's doctors took the more pessimistic view that no good was likely to result from an operation. Thereupon, after reviewing the whole medical evidence and the circumstances, they further found that there was a reasonable certainty that as the result of the operation the man's wage-earning capacity would be restored, that his incapacity might fairly be ascribed to his refusal to undergo the operation, and that such refusal was unreasonable. In consequence of that finding the compensation hitherto paid to the workman was ended.

We conclude by quoting a passage from the opinion of one of the judges which sums up the situation very well: "Nobody says that there is any other way of restoring this man from total incapacity to complete recovery, except by operation, and all the medical men agree that there is neither substantial risk nor prospect of substantial suffering. It seems to me that in ordinary life the appellant would be considered unreasonable by reasonable people."

